# Association of Temporalis Muscle Mass with Early Cognitive Impairment in Older Patients with Acute Ischemic Stroke

**DOI:** 10.3390/jcm12124071

**Published:** 2023-06-15

**Authors:** Ho-geon Namgung, Seungho Hong, Young-Ah Choi

**Affiliations:** Department of Rehabilitation Medicine, Incheon St. Mary’s Hospital, College of Medicine, The Catholic University of Korea, Seoul 06591, Republic of Korea

**Keywords:** cognitive impairment, head MRI, temporal muscle, stroke, sarcopenia

## Abstract

The prognostic value of temporal muscle mass has been studied in various neurological disorders. Herein, we investigated the association between temporal muscle mass and early cognitive function in patients with acute ischemic stroke. This study included 126 patients with acute cerebral infarction aged ≥65 years. Temporal muscle thickness (TMT) was measured using T2-weighted brain magnetic resonance imaging at admission for acute stroke. Within 2 weeks of stroke onset, skeletal mass index (SMI) and cognitive function were assessed using bioelectrical impedance analysis and the Korean version of the Montreal Cognitive Assessment (MoCA), respectively. Pearson’s correlation analyzed the correlation between TMT and SMI, and multiple linear regression analyzed independent predictors of early post-stroke cognitive function. TMT and SMI were significantly positively correlated (R = 0.36, *p* < 0.001). After adjusting for covariates, TMT was an independent predictor of early post-stroke cognitive function, stratified by the MoCA score (β = 1.040, *p* = 0.017), age (β = −0.27, *p* = 0.006), stroke severity (β = −0.298, *p* = 0.007), and education level (β = 0.38, *p* = 0.008). TMT may be used as a surrogate marker for evaluating skeletal muscle mass because it is significantly associated with post-stroke cognitive function during the acute phase of ischemic stroke; therefore, TMT may help detect older patients at a high risk of early post-stroke cognitive impairment.

## 1. Introduction

Stroke and skeletal muscle health deterioration are emerging problems in older adults, and both conditions have been reported to be closely associated with cognitive decline. Depending on the stroke stage, the prevalence of post-stroke cognitive impairment (PSCI) has been reported to be as high as 90% [1,2]. PSCI is common even in the acute phase of minor ischemic stroke (63%) [3]. A prospective study reported that early PSCI was independently associated with poor functional outcomes in the early phase of stroke recovery, and this association was significant even during the convalescent rehabilitation period [4].

Notably, many studies have attempted to elucidate the association between skeletal muscle deficits and poor cognitive performance. A German cross-sectional study that included hospitalized patients showed that loss of lean mass was associated with an increased risk of cognitive deterioration [5]. Furthermore, when comparing healthy older adults with those diagnosed with early-stage Alzheimer’s disease, lean mass was lower in older adults with dementia than in healthy older adults, even after adjusting for sex.

Methods of measuring appendicular skeletal muscle (ASM) include dual-energy X-ray absorptiometry (DXA) and bioelectrical impedance analysis (BIA) [6]. ASM has been used to evaluate skeletal muscle mass after adjusting for body size. However, measuring skeletal muscle mass using DXA or BIA requires high instrument costs and may only be available in some hospitals. Recently, several attempts have been made to use temporal muscle thickness (TMT) as a novel surrogate marker for skeletal muscle mass, risk of sarcopenia, functional outcome, and nutritional status in patients with neurological conditions [7,8,9]. Brain magnetic resonance imaging (MRI) is routinely performed for diagnostic purposes in patients with acute stroke, and TMT measurement is preferred as an easily accessible and alternative method for measuring skeletal muscle mass and nutritional status in patients with acute stroke.

Previous studies have reported the association of early PSCI with mid- and long-term poor functional outcomes [4,10]. A longitudinal study that followed patients with acute ischemic stroke up until 6 months after stroke onset showed that patients with early PSCI were 7.39 times more likely to have a risk of disability than patients with normal cognition [11]. Furthermore, prospective hospital-based cohort studies demonstrated that early cognitive impairment using the Korean version of the Montreal Cognitive Assessment (MoCA) score < 26 within 1 week after stroke onset was associated with worsening cognitive impairment, poor mobility and activities of daily living, and higher mortality risk for up to 3 years after stroke onset [4]. Patients with early PSCI are more likely to be less aware of and less likely to participate in a rehabilitation program, which may reduce the effectiveness of stroke rehabilitation [12,13]. Therefore, screening for early PSCI can provide clinical insights into predicting the overall prognosis after stroke, including post-stroke dementia.

Temporal muscle mass has prognostic significance in various neurological disorders [14]; however, little has been reported about the association between temporal muscle mass and early cognitive function in patients with acute stroke. Therefore, this study aimed to confirm the correlation between TMT and skeletal muscle mass in patients with acute ischemic stroke and whether there is an independent association between temporal muscle mass and early post-stroke cognitive function in older patients. Our study may contribute to the clinical significance of temporal muscle mass in clinical stroke practice.

## 2. Materials and Methods

### 2.1. Study Design and Participants

This mixed retrospective and prospective cohort study included patients with acute cerebral infarction who were hospitalized between 1 January 2020 and 31 December 2022 at the Department of Rehabilitation Medicine in a tertiary hospital. The inclusion criteria were as follows: (1) ≥65 years of age with a premorbid modified Rankin Scale (mRS) score of 0–2; (2) evidence of acute ischemic stroke on brain MRI performed on the day of admission; and (3) BIA measurement and cognitive function evaluation performed within 2 weeks of stroke onset. The exclusion criteria were as follows: (1) inability to measure BIA due to implanted cardiac devices and metal implants; (2) history of neurosurgical or facial surgery before admission; (3) acute delirium; (4) aphasia; and (4) dementia or a major psychiatric disorder diagnosed by a doctor before stroke onset. The institutional review board of our hospital approved this study (OC21ONSI0080). All the methods were performed in accordance with relevant guidelines and regulations. Informed consent was obtained from all the participants.

### 2.2. TMT Measurement Using Brain MRI (T2-Weighted Image)

**Figure 1:**
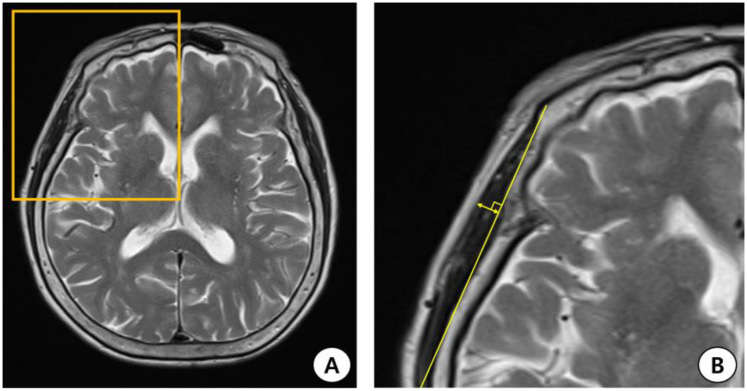
Process of measuring temporal muscle thickness (TMT) on the brain magnetic resonance image (MRI). (**A**) Axial T2-weighted MRI at the orbital roof level. (**B**) The Sylvian fissure is defined as a reference point in the anterior–posterior orientation. TMT was measured perpendicular to the long axis of the temporal muscle.

In this study, two researchers (department of rehabilitation medicine physicians) manually measured TMT using T2-weighted MR images obtained at the time of admission for acute stroke. In the picture archiving and communication system (PACS), the slice thickness was reconstructed to 5 mm. TMT measurements were performed at the orbital roof level in the axial view of the brain MRI. In addition, we placed an axial image parallel to the anterior commissure–posterior commissure line. Furthermore, considering the anteroposterior orientation, the Sylvian fissure was defined as a reference point [15,16,17]. TMT was measured perpendicular to the long axis of the bilateral temporal muscles using the PACS tool (Figure 1). We summed the measured TMT on both sides and divided it by 2 to calculate the average value, which was used for statistical analysis.

### 2.3. Cognitive Evaluation

MoCA was administered within 2 weeks of stroke onset by an experienced occupational therapist who conducted the evaluations under the supervision of certified physicians who specialize in the application of questionnaires and neuropsychological scales. MoCA is known to be feasible for use in patients with acute stroke [18]. The normality cut-off score differs depending on the post-stroke stage. Jaywant et al. (2020) validated the clinical utility of MoCA-defined subgroups for functional outcomes in inpatient stroke rehabilitation, defined as normal (score of 25–30), mildly impaired (score of 20–24), and moderately impaired (score < 19) [19]. Considering the acute phase and the inclusion of older adults, the severity of cognitive impairment was empirically defined based on the literature. The three MoCA-defined subgroups were categorized as follows: ≥20 was classified as normal (score of 25–30) to mildly impaired (score of 20–24); 10–19 was classified as moderately impaired; and 0–9 was classified as severely impaired. An educational correction was applied by adding one point for individuals with ≤6 years of education in the total score calculation [20].

### 2.4. Clinical Examinations

Baseline characteristics were collected, including age, sex, body mass index (BMI), premorbid mRS score, stroke severity measured using the National Institute of Health Stroke Scale (NIHSS) at the time of admission to the emergency room, history of stroke, comorbidities categorized using the Charlson Comorbidity Index (CCI), and risk factors for stroke, including hypertension, diabetes mellitus, atrial fibrillation, and years of education. Imaging information, including stroke lesion side and site, presence of global cerebral atrophy, and Fazekas grade of periventricular hyperintensity (PVH), were obtained from brain MRI at the time of acute stroke onset. The skeletal mass index (SMI) was calculated utilizing the ASM measured using BIA, SMI = ASM/height^2^ (m^2^) [6]. The serum C-reactive protein (CRP) level was measured as a marker of inflammation at the time of admission.

### 2.5. Statistical Analysis

Continuous variables were presented as medians with interquartile ranges (IQRs) (non-normal distribution) or means with standard deviation (SD) (normal distribution). Categorical variables were presented as counts and proportions using percentages. One-factor analysis of variance or the Kruskal–Wallis test for continuous variables and Pearson’s chi-squared or Fisher’s exact test for categorical variables were used to determine whether there were significant differences in the clinical variables between the MoCA subgroups. The inter-rater reliabilities of two observers for TMT were tested using the intraclass correlation coefficient (ICC). ICC values were used to indicate reliability levels as follows: <0.50, poor reliability; 0.50–0.75, moderate reliability; 0.75–0.90, good reliability; and >0.90, excellent reliability [21,22]. Pearson’s correlation analysis was used to evaluate the correlation between TMT and SMI. Multiple linear regression analysis was used to determine whether MoCA scores were independently associated with mean TMT after adjusting for age, sex, BMI, CCI, NIHSS score, years of education, and CRP level. Statistical analyses were performed using R software (R version 4.1.2). Statistical significance was set at *p* < 0.05 (two-tailed).

## 3. Results

### 3.1. Participants and Characteristics

**Figure 2:**
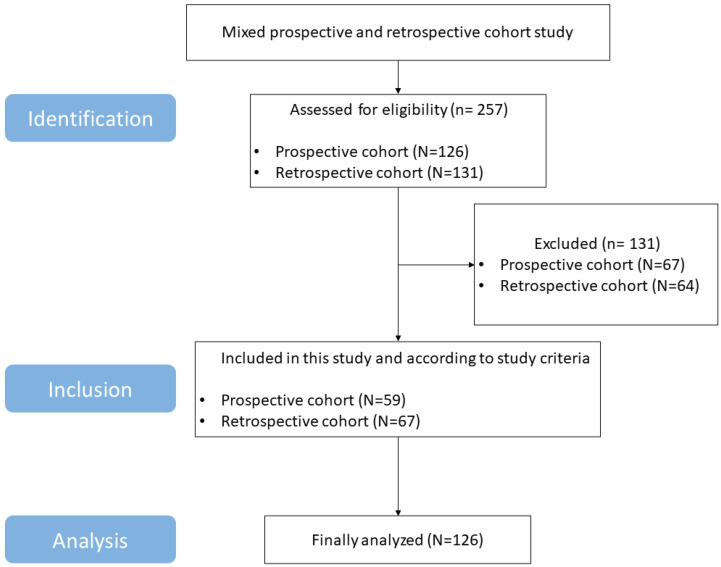
Flow chart of the study.

**Figure 3:**
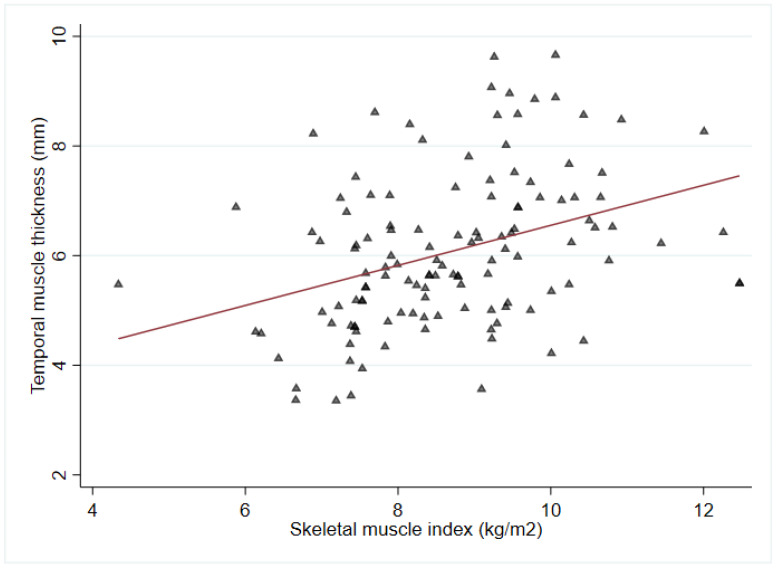
Correlation plot of temporal muscle thickness with skeletal mass index measured using bioelectrical impedance analysis (R = 0.36, *p* < 0.001).

A total of 126 participants (52 males and 74 females; 67 patients from the retrospective cohort and 59 patients from the prospective cohort) were finally included in this study, with a median age of 79 (72–82) years (Figure 2). The median MoCA score was 10 (3.0–17.0), measured within 2 weeks of stroke onset. The median time from hospital admission to transfer to the Department of Rehabilitation Medicine was 9 (6–13) days from the onset date. The average TMT measurement was 6.1 ± 1.4 mm, and TMT measurement had good reliability. The ICCs of right- and left-side TMT were 0.895 (95% confidence interval (CI) 0.853–0.925) and 0.897 (95% CI 0.857–0.927), respectively. The median SMI value was 8.7 (7.6–9.5) kg/m^2^. TMT was positively correlated with SMI (R = 0.36, *p* < 0.001; Figure 3).

### 3.2. Parameters According to the MoCA-Defined Subgroups

**Figure 4:**
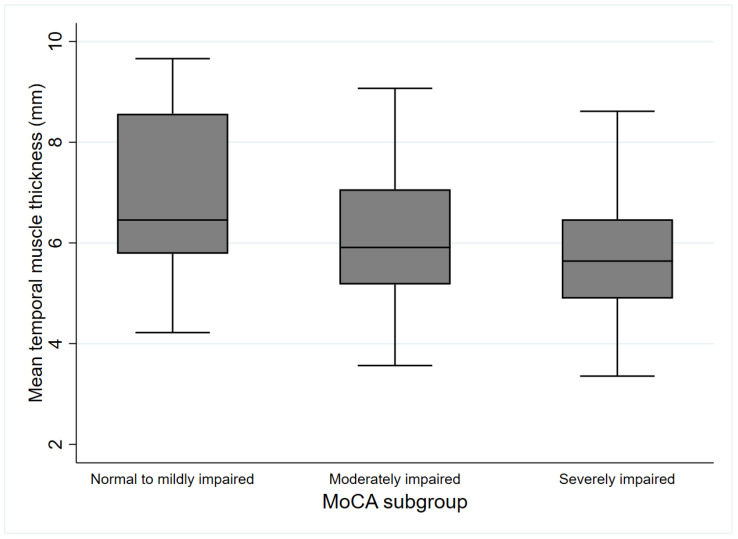
Mean temporal muscle thickness according to MoCA-defined subgroups. MoCA: Montreal cognitive assessment.

Only three patients had a MoCA score ≥25. The clinical variables according to the MoCA-defined subgroup are summarized in Table 1. The median age increased as cognitive function decreased (*p* < 0.001). BMI and the ratio of males were higher in the normal-to-mildly impaired group than in the severely impaired group (*p* = 0.041 and *p* = 0.039, respectively). Stroke severity indicated using the NIHSS was higher in the severely impaired group than in the other two groups (*p* < 0.001). The CCI score was higher in the severely impaired group than in the normal-to-mildly impaired group (*p* = 0.003). Years of education and SMI were higher in the normal-to-mildly impaired group than in the moderately and severely impaired groups (*p* < 0.001 and *p* < 0.001, respectively). The mean TMT values were higher in the normal-to-mildly impaired group than in the severely impaired group (*p* = 0.01; Figure 4). There were no significant statistical differences in the pre-stroke functional status between subgroups categorized using the MoCA. Lesion side and site, white matter diseases evaluated using global cerebral atrophy and Fazekas scale, and the presence of prior stroke were not significantly different among the MoCA-defined subgroups. Furthermore, there were no significant differences in vascular risk factors, including hypertension, diabetes, atrial fibrillation, and CRP levels, among the MoCA-defined subgroups.

### 3.3. Univariate and Multivariate Linear Regression Analyses of Predictors Associated with Early Post-Stroke Cognitive Function

Table 2 shows the results of the univariate and multivariate linear regression analyses for the independent factors associated with early post-stroke cognitive function. No multicollinearity was detected between the independent variables. TMT was independently associated with early post-stroke cognitive function assessed using MoCA scores (β = 1.040, *p* = 0.017), age (β = −0.27, *p* = 0.006), stroke severity (β = −0.298, *p* = 0.007), and education level (β = 0.38, *p* = 0.008).

## 4. Discussion

This study revealed a significant positive correlation between TMT and SMI in older patients with acute ischemic stroke. Even after adjusting for covariates, TMT was an independent predictor of post-stroke early cognitive function indicated using the MoCA score, age, stroke severity, and education level. To the best of our knowledge, this is the first study to report an association between temporal muscle mass on T2-weighted MRI and early post-stroke cognition assessed using the MoCA within 2 weeks of acute stroke.

The clinical risk factors for PSCI include older age, female sex, history of stroke, lower education level, and pre-stroke cognitive impairments [2,23]. Consistent with the literature, older age and lower education level were significantly associated with early post-stroke cognitive decline in the present study. Because infarction size can also affect impairment after stroke, a higher NIHSS score was negatively associated with early cognitive function [24]. The most important finding in the present study was that a lower TMT was independently associated with PSCI even after full adjustment for covariates.

As age advances, the quantity and quality of skeletal muscle change and the possibility of cognitive impairment also increases [25]. Furthermore, there has been notable recognition in the recent literature regarding the significance of the interplay between muscle and cognition. A strong association has been observed between sarcopenia and cognitive impairment in older adults in acute hospital settings. For example, a cross-sectional study involving 619 hospitalized older adults demonstrated that cognitive impairment was independently associated with sarcopenia, even after adjusting for age, sex, nutritional status, activities of daily living, and primary disease [26]. Another German cross-sectional study involving 4095 hospitalized patients showed that loss of lean mass was associated with an increased risk of cognitive deterioration [27]. Similarly, the present study, which included hospitalized older adults with acute ischemic stroke, showed an independent association between TMT and early post-stroke cognitive function.

The existing research suggests a common pathophysiological mechanism underlying skeletal muscle and cognitive decline [28]. For instance, low vitamin D levels, inflammation indicated using interleukin-6 (IL-6) and CRP levels, oxidative damage, low myokine levels (which are muscle-produced factors), and low brain-derived neurotrophic factor (BDNF) levels were negatively associated with cognitive dysfunction. In particular, previous studies have suggested that high levels of inflammatory markers and low BDNF levels may potentially share a pathophysiological link between low skeletal muscle mass and cognitive dysfunction [28,29]. Higher levels of IL-6 and CRP have been associated with skeletal muscle loss [30], whereas changes in molecular biomarkers, such as CRP in the blood, urine, and other body fluids, have been associated with post-stroke cognitive decline [31]. This suggests that individuals with low TMT may be more susceptible to these inflammatory conditions, leading to more severe cognitive impairment after stroke. In addition, BDNF, a neurotrophin growth factor essential for nervous system development and neuronal survival [32], has been associated with frailty and sarcopenia [33], and lower BDNF levels have been observed in patients with stroke [34]. A significantly higher prevalence of cognitive dysfunction has been reported among patients with stroke with low serum BDNF levels than among healthy controls [34]. The correlation between TMT and cognitive function after stroke in the present study can be explained by the hypothesis that underlying biological mechanisms and risk factors may exist between skeletal muscle health and cognitive function. There is a higher likelihood of increased levels of inflammatory markers and decreased BDNF levels in patients with lower TMT. In patients with these fragile characteristics, the occurrence of an acute stroke may further exacerbate higher inflammatory conditions with low BDNF levels, leading to a higher possibility of cognitive impairment.

Physical inactivity and poor diet are well-established common lifestyle risk factors associated with skeletal muscle health deterioration and cognitive impairment [35,36]. Physical inactivity is associated with increased inflammation, anabolic resistance, insulin resistance, and aging. Consequently, blunted muscle protein synthesis and increased muscle protein breakdown are aggravated, resulting in muscle atrophy [37]. Furthermore, physical inactivity decreases cognitive reserve and hampers neuroplasticity, resulting in cognitive dysfunction [38,39]. Malnutrition is closely associated with skeletal muscle loss in geriatric patients undergoing rehabilitation. However, prospective studies have demonstrated that sufficient dietary protein intake protects against sarcopenia. Similarly, previous studies have shown that malnutrition increases the risk of cognitive function deterioration not only in patients with dementia, but also in older adults [40,41]. Furthermore, previous studies have shown that TMT or temporal muscle volume reflects nutritional status [8,42]. The present study included functionally independent patients; however, the quantitative level of physical activity and nutritional status may differ for each patient. We speculated that TMT could comprehensively reflect premorbid physical activity level and nutritional status, which may contribute to the worsening of cognitive function after the occurrence of stroke.

When performing various neuropsychological tests, including the MoCA, ensuring high levels of reliability and validity is crucial. However, performing neuropsychological tests in resource-limited settings, particularly in developing countries, presents challenges [43]. Furthermore, compared with developed countries, older adults in developing countries exhibit a greater degree of educational heterogeneity, with a significantly higher proportion of illiterate individuals and those living in rural areas. Consistent and optimal responses from participants undergoing testing are imperative to ensuring a reliable assessment of cognitive function. However, many individuals may not have prior exposure to test situations in a hospital setting, and illiterate individuals may find it challenging to comply with test instructions [44]. In addition, the lack of trained neuropsychologists in stroke units within developing countries presents a technical challenge. Furthermore, it has been observed that the subject’s performance on screening tests can be significantly impacted by physical disabilities, such as impaired vision or hearing. Vision or hearing impairments, including conditions such as cataracts and presbycusis, commonly coexist in older adults [43]. Therefore, performing cognitive screening tests may not always be feasible in such cases. The present study’s findings suggest that the measurement of TMT obtained from T2-weighted MRI routinely performed for imaging diagnosis of acute ischemic stroke can provide additional information for screening for early PSCI.

In the present study, the positive correlation between TMT and SMI was significant; however, the correlation was relatively weak. Previous studies have consistently reported a positive correlation between TMT and skeletal muscle mass. In a study conducted by Cho et al., the correlation between TMT and ASM was retrospectively evaluated in individuals diagnosed with dementia using cranial MRI and DXA [45]. They found a significant association between TMT and ASM, with an R-value of 0.379 (*p* = 0.001). Another study by Ten Cate et al. investigated the association between TMT and systemic muscle loss in patients newly diagnosed with glioblastoma [46]. They measured skeletal muscle mass at the L3 level using abdominal computed tomography, confirming its correlation with TMT, with an R-value of 0.537 (*p* < 0.001). However, the degree of correlation may vary depending on the imaging modality used for TMT measurement; various equipment for measuring the skeletal muscle mass, such as ultrasonography, DXA, and BIA; or the specific disease being investigated.

Regarding the measurement of muscle parts, skeletal muscle mass is usually measured using the lean mass of the upper and lower extremities. Both the appendicular and craniofacial muscles reflect skeletal muscle mass; however, it can be assumed that the information provided by each muscle part may be clinically different. Considering that several studies have reported that temporal muscle mass has prognostic value in patients with neurological conditions [14], further investigations are required to obtain clinical information on temporal muscle mass, which differs from appendicular muscle mass.

This study has several limitations. First, the sample size was relatively small because this study was conducted in a single hospital. Second, we did not obtain a cut-off value for TMT suggestive of early PSCI. Because our study elucidated the independent association between TMT and early cognitive function after acute stroke, a future prospective study with a sufficient sample size is needed to obtain a cut-off value of TMT for screening early cognitive function. Third, although we thoroughly assessed the patients’ pre-existing cognitive function status and related diagnoses from doctors through detailed interviews and confirmed whether they were taking any relevant medications, we did not administer a structured questionnaire, such as the Informant Questionnaire on Cognitive Decline in the Elderly (IQCODE) [47], to evaluate the pre-existing cognitive function status of all patients. Finally, we evaluated cognitive function using a brief screening instrument within 2 weeks of the acute phase of stroke; however, this may be less applicable in cases of high stroke severity or left hemisphere lesions. Therefore, our study may have included a relatively higher proportion of patients with mild-to-moderate stroke.

## 5. Conclusions

This study confirmed that TMT was positively correlated with SMI measured using BIA in patients with acute cerebral infarction aged ≥65 years. We found that lower TMT, older age, higher stroke severity, and low years of education were independent predictors of early PSCI. TMT measurement can be helpful in detecting patients with acute stroke who may be at a high risk of early PSCI without additional examination or cost burden.

## Figures and Tables

**Table 1:** Baseline characteristics.

MoCA Group	Normal to Mildly Impaired	Moderately Impaired	Severely Impaired	Total	*p*
	(n = 18)	(n = 49)	(n = 59)	(n = 126)	
Age, median [IQR]	71.5 [66.0–74.0]	77.0 [72.0–82.0] *	80.0 [76.5–84.5] *^†^	79.0 [72.0–82.0]	<0.001
Sex, n (%)					0.041
Male	11 (61.1%)	23 (46.9%)	18 (30.5%) *	52 (41.3%)	
Female	7 (38.9%)	26 (53.1%)	41 (69.5%)	74 (58.7%)	
Body mass index, kg/m^2^	24.9 (24.0–27.7)	23.7 (21.4–26.0)	22.9 (20.3–25.6) *	23.6 (20.8–26.0)	0.039
Premorbid mRS score					0.423
0	13 (72.2%)	41 (83.7%)	43 (72.9%)	97 (77.0%)	
1	2 (11.1%)	5 (10.2%)	5 (8.5%)	12 (9.5%)	
2	3 (16.7%)	3 (6.1%)	11 (18.6%)	17 (13.5%)	
Lesion side, n (%)					0.321
Right	9 (50.0%)	28 (57.1%)	25 (42.3%)	62 (49.2%)	
Left	9 (50.0%)	19 (38.8%)	28 (47.5%)	56 (44.4%)	
Bilateral	0 (0.0%)	2 (4.1%)	6 (10.2%)	8 (6.4%)	
Lesion site, n (%)					0.668
Supratentorial	13 (72.2%)	34 (69.4%)	44 (74.6%)	91 (72.2%)	
Infratentorial	5 (27.8%)	14 (28.6%)	12 (20.3%)	31 (24.6%)	
Both	0 (0.0%)	1 (2.0%)	3 (5.1%)	4 (3.2%)	
Stroke severity (NIHSS)	4.0 (2.0–5.0)	3.0 (1.0–6.0)	5.0 (3.0–9.5) *^†^	4.0 (2.0–7.0)	0.001
Cerebral atrophy, n (%)	7 (38.9%)	24 (49.0%)	35 (59.3%)	66 (52.4%)	0.262
Fazekas grade of PVH, grade					0.138
Grade 0	1 (5.6%)	1 (2.0%)	0 (0.0%)	2 (1.6%)	
Grade 1	8 (44.4%)	15 (30.6%)	12 (20.3%)	35 (27.8%)	
Grade 2	7 (38.9%)	23 (47.0%)	27 (45.8%)	57 (45.2%)	
Grade 3	2 (11.1%)	10 (20.4%)	20 (33.9%)	32 (25.4%)	
Recurrent stroke, n (%)	5 (27.8%)	10 (20.4%)	20 (33.9%)	35 (27.8%)	0.297
CCI, point	3.5 (3.0–4.0)	4.0 (3.0–5.0)	5.0 (4.0–5.0) *	4.0 (3.0–5.0)	0.003
Hypertension, n (%)	11 (61.1%)	33 (67.3%)	38 (64.4%)	82 (65.1%)	0.884
Diabetes mellitus, n (%)	6 (33.3%)	19 (38.8%)	26 (44.1%)	51 (40.5%)	0.685
Afib, n (%)	4 (22.2%)	8 (16.3%)	6 (10.2%)	18 (14.3%)	0.385
Year of education, year	12.0 (9.0–12.0)	6.0 (4.0–9.0) *	6.0 (1.5–8.5) *	6.0 (3.0–9.0)	<0.001
MoCA scores, point	22.5 (21.0–24.0)	14.0 (11.0–17.0) *	3.0 (1.0–6.0) *^†^	10.0 (3.0–17.0)	<0.001
SMI, kg/m^2^	9.7 (9.3–10.3)	9.0 (7.9–9.6) *	8.0 (7.4–9.1) *	8.7 (7.6–9.5)	<0.001
Mean TMT (mm)	6.8 ± 1.7	6.2 ± 1.3	5.7 ± 1.3 *	6.1 ± 1.4	0.01
CRP, median [IQR] (mg/L)	2.2 [0.8–7.9]	1.7 [0.7–3.2]	1.9 [0.6–13.4]	1.8 [0.7–6.6]	0.631

IQR: interquartile range; mRS: modified Rankin Scale, NIHSS: National Institute of Health Stroke Scale; PVH: periventricular hyperintensity; CCI: Charlson Comorbidity Index; Afib: atrial fibrillation; MoCA: Montreal cognitive assessment; SMI: skeletal mass index; TMT: temporal muscle thickness; CRP: C-reactive protein. * *p* < 0.05 using post-hoc analyses, comparing moderately and normal-to-mildly impaired groups or severely and normal-to-mildly impaired groups. ^†^ *p* < 0.05 using post-hoc analyses, comparing moderately and severely impaired groups.

**Table 2:** Univariate and multivariate linear regression analyses of the association between clinical variables and early post-stroke cognition function.

Independent Variable	Unstandardized Coefficients			Standardized Beta Coefficients (β)			
	B	SE	*p*	B	SE	*p*	VIF
Age, year	−0.510	0.090	<0.001 *	−0.27	0.097	0.006 *	1.487
Sex [male]	−3.523	1.322	0.009 *	0.154	1.234	0.901	1.329
BMI, kg/m^2^	0.371	0.152	0.016 *	0.189	0.135	0.164	1.227
CCI, point	−1.746	0.416	<0.001 *	−0.512	0.416	0.221	1.415
NIHSS, point	−0.520	0.116	<0.001 *	−0.298	0.109	0.007 *	1.223
History of stroke	−0.912	1.492	<0.001 *	−1.927	1.253	0.127	1.134
Education year, year	0.701	0.138	<0.001 *	0.38	0.140	0.008 *	1.375
CRP, (mg/L)	−0.034	0.027	0.211	−0.034	0.024	0.162	1.208
TMT, mm	−0.910	0.456	<0.001 *	1.040	0.430	0.017 *	1.296

Dependent variable: MoCA scores; R = 0.41 and R^2^ = 0.37; B: beta coefficients; SE: standard error; VIF: variance inflation factor; BMI: body mass index; CCI: Charlson comorbidity index; NIHSS: National Institutes of Health Stroke Scale; CRP: C-reactive protein; TMT: temporal muscle thickness; MoCA: Montreal cognitive assessment. * *p* < 0.05.

## Data Availability

All data generated or analyzed during this study are contained in the article.
